# Antiproliferative and apoptotic effects of xanthohumol in cholangiocarcinoma

**DOI:** 10.18632/oncotarget.21422

**Published:** 2017-09-30

**Authors:** Daniel Walden, Selvi Kunnimalaiyaan, Kevin Sokolowski, T. Gamblin Clark, Muthusamy Kunnimalaiyaan

**Affiliations:** ^1^ Division of Surgical Oncology, Department of Surgery, MCW Cancer Center, Translational and Biomedical Research Center, Medical College of Wisconsin, Milwaukee, WI 53226, USA

**Keywords:** cholangiocarcinoma, xanthohumol, Notch1, apoptosis, cell cycle

## Abstract

Cholangiocarcinoma remains the second most prevalent hepatic neoplasm in the United States with a 5-year survival rate of less than 10%. Currently, no systemic therapy has demonstrated efficacy. Therefore, an urgent need for the identification of molecularly targeted compound(s) remains. The Notch signaling pathway has been shown to be dysregulated in cholangiocarcinoma, exhibiting hyperactivity while also possibly mediating chemotherapeutic resistance. We analyzed the effects of xanthohumol, a prenylated chalcone, on cholangiocarcinoma proliferation utilizing human cholangiocarcinoma cell lines CCLP1, SG-231 and CC-SW-1 while gaining insight into the associated mechanism. Xanthohumol potently reduced cellular proliferation, colony formation, and cell confluency in all three cell lines. Xanthohumol induced cell cycle arrest as well as apoptosis through the reduction of cell cycle regulatory proteins as well as an increase in pro-apoptotic markers (cleaved poly ADP ribose polymerase, cleaved caspase-3) and a decrease in anti-apoptotic markers (X-linked inhibitor of apoptosis and survivin). At the molecular level, xanthohumol reduced Notch1 and AKT expression in a step-wise and time-dependent fashion, with Notch1 reductions preceding AKT. Additionally, xanthohumol reduced cholangiocarcinoma growth in both CCLP-1 and SG-231 derived mice xenografts. In summary, we show that xanthohumol significantly reduced cholangiocarcinoma growth through the Notch1/AKT signaling axis. Furthermore, known pharmacokinetics and bioavailability of XN supports continued development of treatment for cholangiocarcinoma.

## INTRODUCTION

Cholangiocarcinoma (CCA) is characterized by unregulated proliferation of intrahepatic or extrahepatic epithelial cells lining the bile ducts and accounts for 3% of gastrointestinal neoplasms [1]. CCA is the second most prevalent hepatic neoplasm with 6,600 new cases of intrahepatic CCA occurring in the United States in 2014, demonstrating an increase of 3.7% since 2006 [1]. Currently, few therapies exist in the treatment of CCA and the mean survival is 12-months after diagnosis [2]. Poor prognosis is often attributed to the asymptomatic development of the disease, with symptom onset occurring in late-stage CCA, frequently beyond the point of surgical resection [3]. Consequently, the percentage of patients who exhibit a 5-year survival has remained static at 5% over the past 30 years [4]. There is a potential to develop therapies through understanding intracellular pathways that will contribute towards improving survival of CCA patients.

CCA is frequently associated with a predisposition of biliary inflammatory disease. Primary sclerosing cholangitis, a chronic inflammatory biliary tract disease, is the most frequent predisposing factor for CCA [5]. Furthermore, incidence of CCA is significantly higher in Southeast Asia, a region that is endemic to hepatobiliary flukes *Opisthorochis viverrini* and *Clonorichis sinesis* [5]. These liver flukes induce potent inflammatory responses which promotes the production of oxidative stress. [6]. The pro-inflammatory state has been linked to potent pro-survival pathways such as PI3K/AKT, Bcl-2 pathway, and Notch signaling, all of which is shown to be overexpressed in CCA [7].

Notch is a highly conserved trans-membrane protein associated with cell fate and development [8]. Upon ligand activation, Notch is cleaved by γ –secretase, releasing the cytoplasmic domain, which translocates to the nucleus and acts as a transcription factor and regulate various genes expression [8, 9]. Notch enhances proliferation in many different cancers such as bladder, retinoblastoma, and gastric cancer [10–12]. Studies demonstrated that over-activation of Notch signaling in differentiated hepatocytes led to CCA [7, 13, 14]. Cytoplasmic Notch is overexpressed in CCA and is known to promote migration, positively correlate with tumor size (> 5cm), and decrease chemotherapeutic sensitivity [15]. Inhibition of Notch induces cell cycle arrest and apoptosis in CCA [16]. We suspect that targeting Notch in CCA may drastically inhibit the oncogenic phenotype, allowing us to better understand and treat CCA in future studies.

Xanthohumol (XN), a prenylated chalcone derived from *Humulus lupulus*, is a novel compound that has been shown to be efficacious in the prevention of carcinogenesis and neoplastic development [17]. Initial studies using XN on CCA cell lines have shown that a 10 μM doses of XN induces a 5-fold reduction in prostaglandin E2 (PGE2), a product predominantly found to promote CCA migration [17]. We have previously shown that XN can reduce Notch1 levels in human hepatocellular and pancreatic carcinoma [18, 19]. However, no studies have shown the anti-carcinogenic efficacy of XN in human CCA cell lines. Here we sought to investigate the *in vitro* and *in vivo* effect following XN treatment in CCA.

## RESULTS

### Xanthohumol inhibits proliferation, colony formation, and cell confluency in CCA

**Figure 1:**
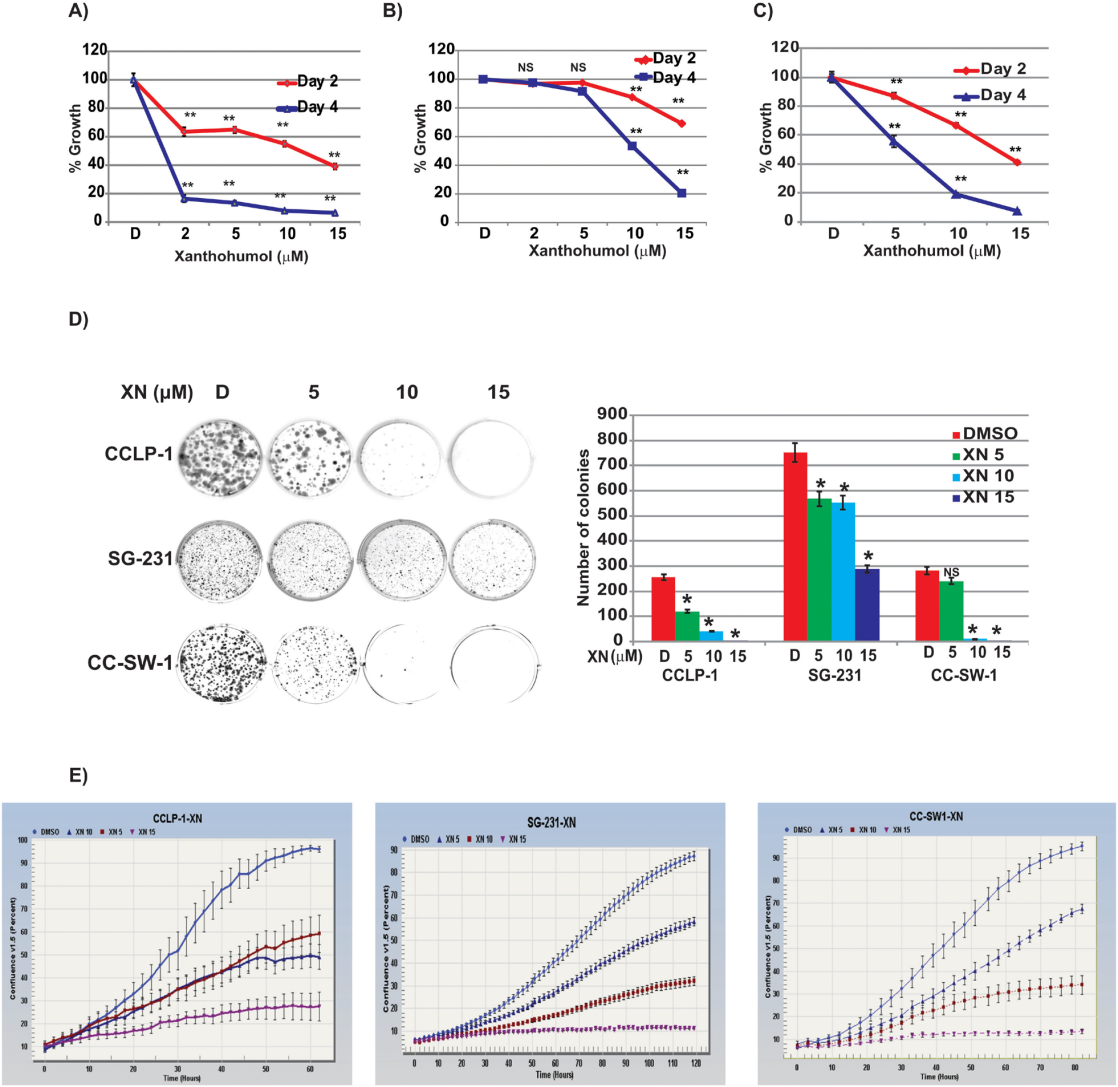
Effects of XN on CCLP-1, SG-231, and CC-SW-1 cellular proliferation **(A)** Cells were treated with indicated concentrations of XN and cell proliferation was measured by MTT assay. Statistically significant (p<0.001) (^**^) growth suppression was observed in CCLP-1 (A), SG-231 **(B)**, and CC-SW-1 **(C)** cells when treated with XN in both 2 and 4-day treatments (NS, not significant). **(D)** XN treatment inhibits the clonogenic ability of CCA cell lines in colony formation assay. Bar graph shows the number of colonies formed after XN treatment **(E)** Cells were treated with indicated concentrations and cell proliferation was monitored in real time with the continuous presence of XN. The cells were photographed and the cell confluence was calculated using IncuCyte 2011A software. The changes in cell confluence was used as surrogate marker of cellular proliferation. Significant growth suppression was observed with increasing concentrations of XN. These were statistically significant.

Cellular viability of CCA cell lines was assessed via 3-(4, 5-dimethylthiazol-2-yl)-2, 5-diphenyltetrazolium bromide colorimetric assay (MTT). CCLP-1, SG-231, and CC-SW-1 exhibit concentration and time dependent growth reductions to XN after 48 and 96 hours (Figure 1A, 1B, 1C respectively). CCLP-1 cells (1A) treated for two days at 5, 10, and 15 μM concentrations of XN exhibited a reduced viability of 33%, 42%, and 57%, and for four days exhibited reduced viability of 56%, 60%, and 84% respectively compared to control. Statistically significant (p-value <0.001) values were seen at all three concentrations. SG-231 (1B) had reduced viability after two days of exposure to XN corresponding to 2%, 13%, and 31% and at 4 days 8%, 47%, and 80% at 5, 10, and 15 μM respectively. Statistically significant (p-value <0.001) values were obtained at 10 and 15 μM concentrations. CC-SW-1 showed 13%, 33%, and 59% at 5, 10, and 15 μM respectively at day two whereas increased reductions were observed at day 4 (44%, 81%, and 93%) compared to control (1C). To confirm the anti-proliferative properties of XN, CCA cells were plated to determine their ability to form colonies after XN-treatment. As shown, XN dramatically inhibits the ability of CCLP-1, SG-231, and CC-SW-1 cells to form viable colonies (Figure 1D). Following administration of XN, the ability of CCA cell lines to form colonies is greatly reduced even at the lower concentrations (5 μM) tested. Administration of XN resulted in a dose and time-dependent inhibition of cellular proliferation as well as colony formation. Increased concentrations of XN reduced the confluency of all three cell lines in a time and dose-dependent manner as measured by the cell confluence real-time kinetics (Figure 1E).

### Xanthohumol induces cell cycle arrest through reduction of cyclin D and its binding partners CDK

**Figure 2:**
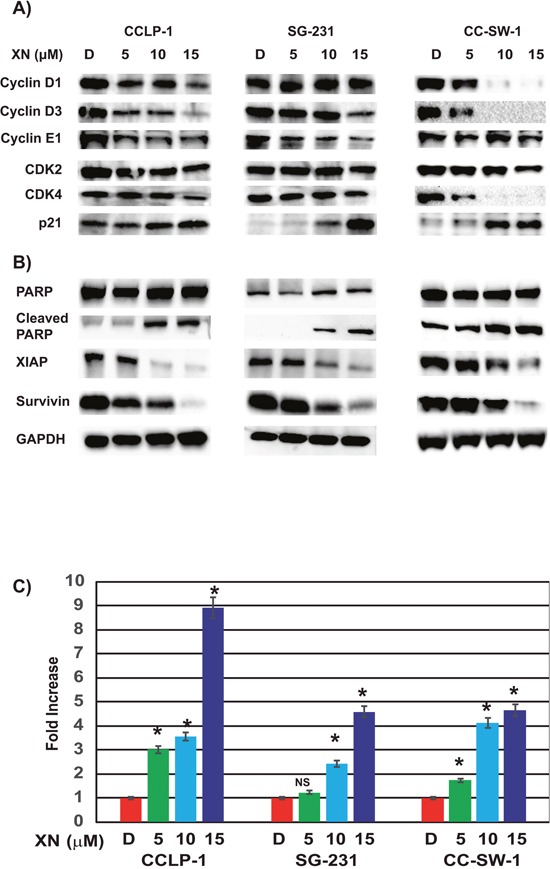
XN induces cell cycle arrest and apoptosis in CCA cell lines **(A)** Treatment with XN increases p21, cyclin-dependent kinase inhibitor, in all three cell lines. Key cell cycle regulators cyclin D1, Cyclin D3, Cyclin E1, and CDK2 were reduced in CC-SW-1 whereas cyclin D3 and cyclin E1 and CDK2 and CDK4 were reduced at various levels in CCLP-1 and SG-231. **(B)** XN-treatment induces apoptosis as evidenced by the increase in cleaved PARP in three cell lines. Furthermore, pro survival protein survivin as well as anti-apoptotic protein XIAP was reduced with XN-treatment. Glyceraldehyde 3-phosphate dehydrogenase (GAPDH) is shown as a loading control. **(C)** Caspase-3 and -7 activities were measured by caspase Glo3/7 assay (^*^, p<0.05 compared to control in three cell lines tested; NS, not significant).

To determine the etiology of the growth suppressive nature of XN, we analyzed the quantity of cell cycle markers in three CCA cell lines. CCLP-1 cells showed reductions in cyclin D3, cyclin D1, cyclinE1, and CDK4 suggesting that XN treatment prevents cell cycle progression through the G1 phase (Figure 2A). SG-231 displayed XN induced reductions in cyclin D3, cyclin E1, as well as in CDK4 (Figure 2A). In CC-SW-1 cells, XN treatment reduced the levels of cyclin D1, cyclin D3, and CDK4; suggesting that XN causes cell cycle arrest at the G0/G1 phase of the cell cycle (Figure 2A). Additionally, all three cell lines elicited an increase in p21 expression. These findings suggest the observed growth restriction and subsequent cell death is caused by cell cycle arrest and the induction of apoptosis.

### Xanthohumol induces apoptosis in CCA cells

When there is a delay in DNA repair during the cell cycle check point, cells undergo apoptosis. Western analysis revealed that all three cell lines expressed an increase in cleaved poly ADP ribose polymerase (PARP) and a reduction in the pro-survival protein, survivin indicating apoptosis (Figure 2B). Additionally, all three cell lines showed reductions in X-linked inhibitor of apoptosis protein (XIAP) expression in response to XN-treatment. CCLP-1 showed a significant reduction in XIAP following 5 μM XN concentrations, while SG-231 and CC-SW-1 exhibited a gradual, dose-dependent suppression. These apoptotic results were confirmed by luminescence assay which measures caspase-3 and -7 activities. As shown in Figure 2C, there was a significant increase in luminescence with increasing concentrations of XN treatment. 15uM concentrations of XN caused 9, 5, and 5-fold increased luminescence for CCLP-1, SG-231, and CC-SW-1 respectively. Taken together, these results suggest XN reduces cell proliferation via cell cycle arrest and eventual apoptosis.

### Xanthohumol induced alterations in Notch1/AKT signaling pathways

**Figure 3:**
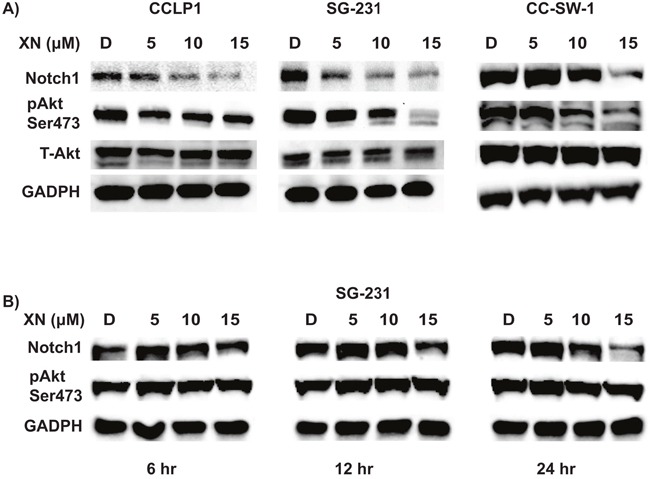
XN treatment alters Notch1 and PI3-K/AKT pathway **(A)** Dose-dependent reductions in Notch 1 in CCLP-1, SG-231, and CC-SW-1 correspond to increasing dose of XN were observed after 72 hours of XN treatment. Phosphorylated AKT at serine 473 (p-Akt Ser 473) is reduced when compared to total cellular AKT. Glyceraldehyde 3-phosphate dehydrogenase (GAPDH) is shown as a loading control. **(B)** Time course experiments in SG-231 showed Notch1 reduction at 12 hr after XN treatment whereas no reduction in phosphorylated AKT at ser473 position even after 24 hr treatment. Glyceraldehyde 3-phosphate dehydrogenase (GAPDH) is shown as a loading control.

Previously we have shown that XN reduces Notch1 in pancreas and hepatocellular cancer [18, 19]. To evaluate the mechanism of reduced cellular viability of XN-treated CCA cells, Western blot analysis of cell regulatory signaling pathways was performed. Western blot analysis revealed CCLP-1, SG-231, and CC-SW-1 exhibited dose-dependent reductions in Notch1 compared to control treatment (Figure 3A). Furthermore, Notch reduction was associated with reduction in phosphorylation of AKT, a key downstream mediator of the PI3-K/AKT pathway (Figure 3A).

### Reduction in Notch1 expression preceded phosphorylated AKT reduction

Determining whether reduction in Notch1 preceded, occurred concurrently, or because of decreased AKT phosphorylation is critical to evaluate. We carried out western analysis of SG-231 cells treated with XN at various time points to delineate this issue. As shown in Figure 3B, reduction in Notch1 was observed as early as 12 hrs after XN treatment. At 24hrs the Notch1 was further reduced. There was no reduction of phosphorylated AKT at 12 hrs or even at 24 hrs after treatment suggesting that Notch1 reduction precedes AKT phosphorylation.

### Xanthohumol treatment reduces tumor growth in mouse xenograft model

**Figure 4:**
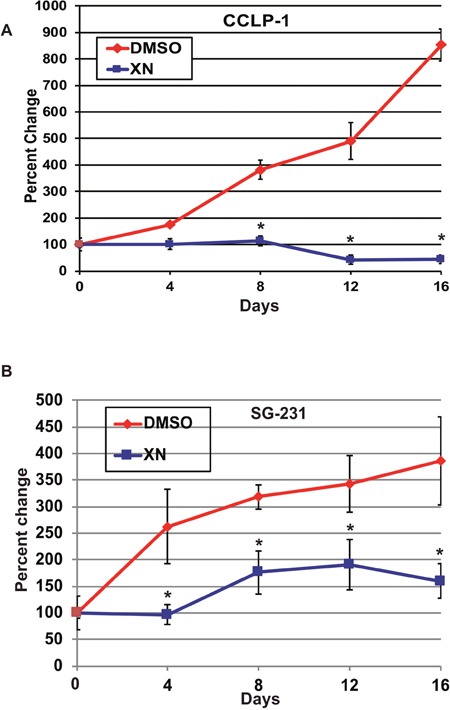
XN treatment inhibits tumor growth in both CCLP-1 and SG-231 cells injected in a mice xenograft model **(A)** CCLP cells 8 days after treatment control mice demonstrated 381% change in tumor volume compared to a XN treated mice which showed a 13% decrease in tumor volume. After 16 days, control mice had a tumor volume 853% larger than on day 0, compared to XN treated mice, which had a 78% reduction in tumor volume. **(B)** SG-231 cells 8 days after injection untreated mice showed a 427%-fold increase in tumor size compared to a 153% change in XN treated mice. After 16 days, control and XN treated mice demonstrated 1308% and 255%-fold changes, respectively. CCLP-1 developed tumor did not increase in percentage fold after XN treatment throughout the treatment time points where as in SG-231 tumors there was increase in tumor fold and maintained the level during treatment compared to control treatment. Data were represented as +/− s.e.m. ^*^, p <0.001.

To examine the effect of XN treatment *in vivo*, we performed a xenograft study using CCLP-1 and SG-231 cell lines in an immunodeficient, athymic mouse model. Cells were injected subcutaneously into the flank of athymic nude mice. The mice were intraperitoneally injected with XN at 125 μg per mouse (5 mg/kg body wt of approximately 25g mouse) every other day for 16 days. In both cell lines, the XN treatment reduced tumor growth significantly (Figure 4A and 4B). In mice injected with SG-231 cells, tumor burden was substantially restricted. 8 days after injection untreated mice showed a 427%-fold increase in tumor size compared to a 153% change in XN treated mice. After 16 days’ control and XN treated mice demonstrated 1308% and 255%-fold changes, respectively. Additionally, CCLP-1 injected mice demonstrated tumor shrinkage as a result of XN treatment. After 8 days of treatment control mice demonstrated 381% change in tumor volume compared to a XN treated mice which showed a 13% decrease in tumor volume size. After 16 days, control mice had a tumor volume 853% larger than on day 0, compared to XN treated mice, which had a 78% reduction in tumor volume. Here we show that, *in vivo*, XN not only has the capacity to reduce the growth of CCA, but also the ability to actively decrease tumor burden.

## DISCUSSION

Cholangiocarcinoma is a disease characterized by limited symptomatology and aggressive metastasis leading to significantly high mortality. Consequently, there is a low possibility of surgical intervention [3]. Surgical resection remains the only curative treatment for CCA, yet few are candidates due to late stage presentation. Surgical candidates unfortunately have a poor prognosis, as they are typically limited to a 12-15-month median survival rate [20]. Thus, the need for advanced treatment, early diagnosis, and cellular understanding of CCA is critical.

Notch1 signaling is associated with proliferation, chemoresistance, metastasis, tumorigenesis and is overexpressed in many organ-specific cancers including liver and cholangiocarcinoma [21, 22]. Additionally, Notch1 is an important regulator of cellular proliferation in cancer stem cells, a small subset of cancer cells known to promote the tumor microenvironment and drive tumorigenesis [23]. Recently, Notch1 inhibition has shown to deplete the cancer stem cell population in gastric, breast, and glioma cancers through inhibiting angiogenesis and sensitizing the tumors to chemotherapy [23–25]. Clinical trials targeting Notch are currently ongoing. A new γ-secretase inhibitor, MK-0752, is currently in a phase I clinical trial for patients with pancreatic cancer. Primary results show there is a therapeutic benefit when given with gemcitabine in 11 out of 18 patients exhibiting progression free disease and confirmed partial response (NCT01098344). Additionally, a novel histone deacetylase with anti-Notch1 characteristics is currently in a phase I clinical trial as a potential therapy for medullary thyroid cancer (NCT01013597).

In this study, our results have confirmed that xanthohumol reduces Notch1 expression leading to apoptosis. Our data suggests that XN inhibits proliferation of CCA in both a dose and time dependent manner. The reduction of Notch1 levels after treatment in CCA is consistent with our previous data on hepatocellular carcinoma and pancreatic cancer [18, 19]. Therefore, we believe that Notch1 reduction is important for growth suppression by XN in CCA. Furthermore, Notch inhibition elicited the reduction in phosphorylation of AKT at the serine 473 residue without changing the levels of total AKT. AKT is overexpressed in roughly 70% of CCA cases and is strongly implicated in promotion of carcinogenesis in many cancers [26, 27]. Furthermore, inhibition of AKT by LY294002 significantly suppressed human-growth factor-stimulated invasion in CCA by stabilization of E-cadherin [28]. Recently, we have shown that inhibition of the AKT pathway by MK-2206, AKT inhibitor, resulted in growth suppression [29].Therefore, we speculate that inhibition of both Notch and AKT pathways should be synergistic and more efficacious than inhibition of either pathway alone.

Our data provides evidence that XN inhibits human CCA (CCLP-1, SG-231, CC-SW-1) proliferation and induced apoptosis in both *in vitro* and *in vivo* models. We also demonstrated that XN inhibits Notch1 signaling as early as 12hrs after treatment. Reduction in phosphorylation of AKT at 3 days suggested that Notch signaling precedes and may initiate cross-talk with the PI3K/AKT signaling pathway. Furthermore, *in vivo* mice studies demonstrated inhibition of tumor progression with XN treatment.

Recent evidence into xanthohumol anti-tumorigenic abilities has been documented in various organ-specific cancer cell lines. Pharmacological studies suggest a large therapeutic window for XN and given a lower toxicity profile at lower concentrations, potential adverse effects are mitigated [30]. Such evidence suggests that XN could potentially be a novel agent for CCA. Our data warrants continued research into the efficacy of XN as a treatment strategy or adjunct therapy for CCA.

## MATERIALS AND METHODS

### Cell Culture and XN Treatment

Human CCA cell lines (CCLP-1 and SG-231), derived from intrahepatic biliary epithelium, were obtained from Dr. Anthony J. Demetris from the University of Pittsburgh. The CC-SW-1 cell line was kindly provided by Dr. Nabeel Bardeesy, Harvard Medical School. All cell lines were authenticated before receiving them. CCLP-1 cells were grown in Dulbecco Modified Eagle Medium (DMEM; Sigma-Aldrich, St. Louis, MO) with 1% penicillin/streptomycin (P/S; Life Technologies, Carlsbad, CA), 10% fetal bovine serum (FBS; Life Technologies), 1% 4-(2-hydroxyethyl)-1-piperazineethanesulfonic (HEPES; Life Technologies), and 1% non-essential amino acids (NEAA; Life Technologies). SG-231 cells were grown in Minimal Essential Medium alpha-1 (MEMα-1; Life Technologies) with 10% FBS, 1% P/S, and 1% HEPES [16]. CC-SW-1 cells were grown in Roswell Park Memorial Institute (RPMI) 1640 medium with 10% FBS and 1% P/S. Cells were grown in a 37°C humidified incubator in 5% CO_2_. Xanthohumol (Tocris, Minneapolis, MN or Selleckchem.com, Houston, TX) was dissolved in dimethyl sulfoxide (DMSO; Sigma-Aldrich).

### Cellular viability assay

CCLP-1, SG-231, and CC-SW-1 cellular viability was measured through 3-(4, 5-dimethylthiazol-2-yl)-2, 5-diphenyltetrazolium bromide colorimetric assay (MTT). Cells were plated onto 96-well plates and allowed to adhere overnight. XN was added in 5, 10, and 15 μM concentrations in triplicate. The control wells were treated with DMSO. Cells were incubated for either 48 or 96 hours. Media was replaced with 50 μL RPMI1640 containing 0.5 mg/mL MTT and incubated for 3 hours. Following incubation, 150 μL of DMSO was added to each well. Cellular absorbance was measured at 540 nm (Infinite M200 Pro; Tecan, San Jose, CA). Absorbance values were then calculated as percent cellular viability by dividing absorbance of the respective treatments groups by the control group. Statistical analysis was performed using the online software GraphPad (La Jolla, CA). Unpaired t-tests were performed on each data set and P-values <0.05 were considered significant.

### Colony formation assay

To further assess proliferation and efficacy of XN, colony formation assay was performed on these three cell lines. Cells were plated onto 6-well plates and allowed to adhere overnight. Cells were treated with DMSO (control), 5, 10, or 15 μM concentrations of XN for three days and then media was changed every 3 days without XN for 2 weeks. Cells were then fixed with crystal violet and images were obtained with Molecular Imager Chemi-Doc XRS^+^ imager with Image Lab software (Bio-Rad, Hercules, CA).

### Non-invasive cellular proliferation assay in real time

Using IncuCyte Live-Cell imaging system (Essen Bioscience), cellular proliferation of CCA cell lines were measured as described [19]. Cell confluence was calculated using IncuCyte 2011A software and the cell proliferation was expressed as an increase in percentage of confluence with different time intervals.

### Western blot analysis

Cell lysates were obtained following 3 days of XN-treatment using the radioimmunoprecipitation assay buffer (RIPA; Thermo Fisher Scientific. Waltham, Mass). Protein quantification was measured by BCA protein assay (Thermo Scientific). Thirty micrograms of protein were loaded on to 7.5%, 10%, or 12% sodium dodecyl sulfate polyacrylamide gels (Bio-Rad Laboratories). Gels were run until the proteins separated and were transferred onto a nitrocellulose membrane using the Trans-Blot Turbo Transfer System (Bio-Rad). Transferred membranes were blocked in a 5% dry milk solution and incubated in primary antibody overnight at 4°C. Primary antibodies used were: Notch1 (1:1000), Survivin (1:500), Cyclin D1 (1:500), total and phosphorylated AKT (1:1000), p21 (1:200), total poly-ADP ribose polymerase (PARP) (1:2000) and glyceraldehyde 3-phosphate dehydrogenase (GAPDH) (1:4000) all from Santa Cruz Biotechnologies, Dallas, TX), cleaved PARP, X-linked inhibitor of apoptosis protein (XIAP), Cyclin D3, Cyclin E1, CDK2, and CDK4 all (1:1000) from Cell signaling Technology, Boston, MA. After overnight incubation, membranes were washed three times in a 1x phosphate buffered solution with 0.05% Tween-20 buffer. Following membrane wash, the blots were incubated with horse-radish peroxidase linked anti-mouse or anti-rabbit secondary antibody (1: 5,000, Santa Cruz) depending on the source of the primary antibody. The membranes were developed using Luminol (Santa Cruz) Supersignal West Dura or West Femto (Thermo Fisher Scientific). The membranes were then imaged using the Molecular Images Chemi-Doc XRS^+^ imager (Bio-Rad).

### Caspase-3 and -7 activities

Caspase-Glo 3/7 Assay (Promega, Madison, WI) kit was used to measure the active caspase 3 and -7 from the lysates of cells treated with XN as described [19]. Luminescence was measured using Infinite M200PRO Microplate reader (TECAN).

### Cholangiocarcinoma tumor xenografts in mice

CCLP-1 or SG-231 (1 × 10^6^ cells in 100 μl) were subcutaneously injected into the flank of 6-week-old BALB/c nude mice obtained from the Charles laboratory. Treatments (125 ug of XN dissolved in saline was injected intraperitoneally into the treatment group every other day) were started after palpable tumors were seen. The mice were divided into two groups (n = 5) and treated with either control (saline) or XN in saline for every other day for a total of 16 days. Mice tumors were measured every 4 days and the tumor volume was calculated using the formula X^2^Y/2, where x is the smallest and y is the largest diameters. Percentage of fold change was calculated.
